# The Anatomist James Douglas (1675-1742): His Life and Scientific Work

**DOI:** 10.7759/cureus.3919

**Published:** 2019-01-19

**Authors:** Konstantinos N Koutsouflianiotis, George K Paraskevas, Nikoleta Kalitsa, Kalliopi Iliou, George Noussios

**Affiliations:** 1 Internal Medicine, General Hospital of Thessaloniki "G. Gennimatas", Thessaloniki, GRC; 2 Orthopaedics, Aristotle University of Thessaloniki, Thessaloniki, GRC; 3 Miscellanoeus, University of Ioannina, Thessaloniki, GRC; 4 Psychiatry, Aristotle University of Thessaloniki, Thessaloniki, GRC; 5 Otolaryngology, Aristotle University of Thessaloniki, Thessaloniki, GRC

**Keywords:** douglas, anatomy, pouch, recto-uterine, peritoneum

## Abstract

James Douglas (1675-1742) is considered one of the most important anatomists of the eighteenth century; he introduced meticulous and scientific methods for studying human anatomy. He is known for the “pouch of Douglas,” but his contribution is much more important. He deepened our knowledge of the anatomy of the peritoneum, located new muscles, and evolved the already recorded knowledge in a way that it could be implemented in surgery. Furthermore, he was such a famous obstetrician that even the Pope of his era admired him for his charisma.

## Introduction and background

James Douglas (1675-1742), born at Badds, near Edinburgh, was one of a 12-child family, including the well-known lithotomist John Douglas. His father was an important landowner in the region and of some social standing. James Douglas studied at Edinburgh University where he received his Masters of Arts degree in 1694. It is not known where he received his medical training, but he received his medical degree from Reims University in 1699 [1-2].

## Review

In 1700, he arrived in London, where he worked with Paul Chamberlen, a famous obstetrician. Douglas treated Chamberlen’s patients in his absence and received advice from him on therapies. Douglas’ patients at this time were ordinary people, mainly small traders or craftsmen. He recorded meticulously their symptoms, made his own observations, and documented the results of his treatment. As time went by, he continued treating the poor but gradually started treating the aristocracy too. His treatment, however, like that of almost all his contemporaries, was basically still Galenic, relying on the balance of the humors, causing vomiting by prescribing herbs of unknown specific action. If his patients died, he generally sought permission to carry out a necropsy [2].

James Douglas was a famous anatomist, gynecologist, and a fine botanist and zoologist, whose collection of the works of Horace was matchless. Douglas was the mentor of William Hunter and when the last died in 1783, demised his collections to Glasgow University, which formed the basis of the Hunterian Library and Museum. The documents comprise manuscripts written by James Douglas while almost all of them remain unpublished [3]. Douglas who was looking for a dissector, not only hired Hunter but also assigned him as an instructor of one of his sons. Through Douglas’s acquaintances, Hunter became a surgical assistant at St. George’s Hospital. Douglas was impressed by Hunter’s capabilities but their acquaintance was interrupted, as Douglas died in Spring 1742. However, this did not end Hunter’s connection with the Douglas family with whom he continued to live until 1749. He was probably engaged to Douglas’s daughter but she died in 1744 [4].

Douglas conducted private lectures of anatomy in London, for, in 1707, he was advertising “An Account of What Dr Douglas Obliges Himself to Perform in a Course of Human and Comparative Anatomy.” This systematic course was given three or four times yearly, at a charge of three and a half guineas [3]. The reason Douglas repeated the course several times is firstly financial. Secondly, Douglas was teaching something different than what surgeons taught in Surgeons’ Hall. He focused on comparative anatomy and not on surgical techniques, as was occurring in the practically oriented courses for surgeons. Douglas utilized in his course anatomical preparations as well as “fresh bodies.” When his cadavers became dry, the bodies could be further refreshed by injecting arteries and other vessels with colored wax or substances such as mercury. He spent much time perfecting his injection techniques [5].

Douglas was being elected since 1706 as a Fellow of the Royal Society and his election was followed by very active participation in its meetings. He made over 40 original communications to the Society, 14 of which were published in the Philosophical Transaction. In 1712, he was elected to the Gale Osteology Lectureship of the Barber-Surgeons Company and to the Arris Muscular Lectureship in 1716. In 1720, Douglas became Honorary Fellow of the Royal College of Physician, although he was not a graduate of Oxford or Cambridge [2].

**Figure 1:**
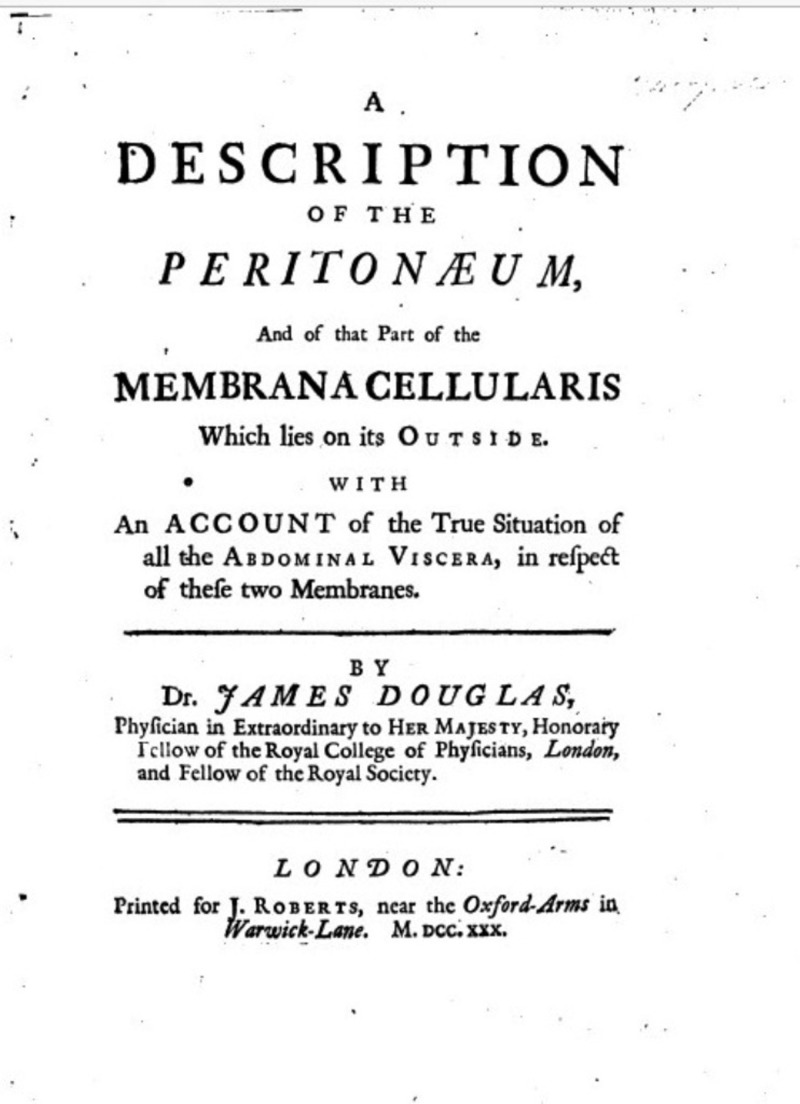
The cover page of the famous book by James Douglas named “A Description of the Peritoneum, and of that Part of the Membrana Cellularis which lies on its Outside, with an Account of the True Situation of all the Abdominal Viscera” published in 1730. In this book, for the first time, is recorded the famous “pouch of Douglas.” Public domain.

In 1707, Douglas published his anatomical textbook “Myographiae Comparatae Specimen, or a Comparative Description of all the Muscles in a Man and in a Quadruped.” In this book, Douglas displayed 13 muscles as his own discoveries, but it is worth mentioning that none of these has achieved eponymic recognition. On the other hand, it is impressive that the “semilunar fold” and the “line of Douglas” have not been found anywhere described [3]. It has to be mentioned that Douglas in his book, “A Description of the Peritoneum and the Part of the Membrana Cellularis which lies on its Outside, with an Account of the True Situation of all the Abdominal Viscera” that was published in 1730 (Figure 1), described the “line of Douglas,” defining it indirectly. Specifically, he wrote, “This being done (a longitudinal incision), we find the peritoneum closely connected to the tendon of the transversalis, scarce any vesicular substance being perceivable by the naked eye between them; and therefore a great deal of nicety and patience is required in dividing this tendon from the peritoneum, all the way to the fleshy bellys on each side. I next go on to the lower part of this foreside, where the musculi recti come between the tendon of the transversalis and peritoneum; and here the separation is easily made, because the quantity of cellular substance increases considerably all the way down to the os pubis” [6].

The other eponymic features are the “pouch of Douglas” and the “ligaments of Douglas,” thus the peritoneal folds that limit it laterally [3]. In his book regarding the peritoneum, Douglas described the pouch named after him. Through anatomy, James Douglas made an important contribution to surgery. In particular, he stated: “Where the peritoneum leaves the foreside of the rectum, it makes an angle and changes its course upwards and forward over the bladder. A little above this angle there is a remarkable transverse stricture or semi-oval fold of the peritoneum which I have constantly observed for many years past, especially in women” [7]. The remarkable stricture or fold, the first description of which Douglas credits to Winslow, was at one time called the ligament of Douglas but is now known as the recto-uterine fold [2]. The reason for studying the peritoneum is answered by James Douglas in the same book. “When I began my inquiries about this important membrane, I had the etiology of several diseases principally in view; among which were dropsys, hernias, and some other accidents peculiar to women.” And he continued “upon the revival of the high operation for the Stone, by my brother the surgeon, I likewise undertook to consider the peritoneum with the relation to the different methods of Lithotomy for the safe performance of which knowledge of this membrane is of the utmost importance” [8].

As early as 1713, Douglas had been working on his “Osteographia” and fine artists, such as Francois Boitard, were employed in order to complete the ambitious project. In 1729, Douglas exhibited at the Royal Society a set of 47 figures and their descriptions and later replanned the figures, now numbering over 60. The current study, which was supported by King George I, was never published, although the biggest part of the work was finished. If it had been published, undoubtedly, it would have been the greatest anatomical work of the eighteenth century [2].

## Conclusions

James Douglas died in 1742 and William Hunter gave a picture of the death of James Douglas while writing to his mother. “Early on the morning of Dr Douglas’s death, I was called at his desire; he snatched at my hand and spoke a few words; he would not let me go out of the room; I sat on his bed till after noon when he expired with his hand locked in mine.” James Douglas’s legacy is to be remembered for his penetrating scientific mind, his profound dedication to the relief of patients, and his passion for the evolution of knowledge. Even the Pope showed his appreciation of the great physician in the following lines: “There all the learn’d shall at the labour stand, and Douglas lend his soft obstetric hand.”

## References

[1] Persaud TVN, Loukas M, Tubbs RS (2014). A History of Human Anatomy.

[2] Brock H (1974). James Douglas of the pouch. Med Hist.

[3] Thomas BK (1960). James Douglas of the pouch, 1675-1742. Br Med J.

[4] Donaldson IM (2016). Smellie & Hunter: atlases of the gravid uterus. Part 2. J R Coll Physicians Edinb.

[5] Guerrini A (2004). Anatomists and entrepreneurs in early eighteenth-century London. J Hist Med Allied Sci.

[6] Douglas J (1730). A description of the peritoneum, and of that part of the membrana cellularis which lies on its outside, with an account of the true situation of all the abdominal viscera. https://books.google.gr/books?id=ZKpbAAAAcAAJ&pg=PA35&dq=james+douglas+peritoneum&hl=en&sa=X&ved=0ahUKEwjR9_TV3djfAhUQJ1AKHcjqAVsQ6AEIPzAF#v=onepage&q=james%20douglas%20peritoneum&f=false.

[7] Douglas J (1730). A description of the peritoneum, and of that part of the membrana cellularis which lies on its outside, with an account of the true situation of all the abdominal viscera. https://books.google.gr/books?id=ZKpbAAAAcAAJ&pg=PA35&dq=james+douglas+peritoneum&hl=en&sa=X&ved=0ahUKEwjR9_TV3djfAhUQJ1AKHcjqAVsQ6AEIPzAF#v=onepage&q=james%20douglas%20peritoneum&f=false.

[8] Oughterson AW (1930). James Douglas and the surgery of the peritoneum. Yale J Biol Med.

